# The Effects of the ManageHF4Life Mobile App on Patients With Chronic Heart Failure: Randomized Controlled Trial

**DOI:** 10.2196/26185

**Published:** 2021-12-07

**Authors:** Michael P Dorsch, Karen B Farris, Brigid E Rowell, Scott L Hummel, Todd M Koelling

**Affiliations:** 1 Department of Clinical Pharmacy College of Pharmacy University of Michigan Ann Arbor, MI United States; 2 Frankel Cardiovascular Center University of Michigan Ann Arbor, MI United States; 3 Division of Cardiovascular Medicine Medical School University of Michigan Ann Arbor, MI United States; 4 Ann Arbor Veterans Affairs Health System Ann Arbor, MI United States

**Keywords:** mHealth, remote monitoring, self-management, self-care, heart failure, medical therapy, mobile app

## Abstract

**Background:**

The successful management of heart failure (HF) involves guideline-based medical therapy as well as self-management behavior. As a result, the management of HF is moving toward a proactive real-time technological model of assisting patients with monitoring and self-management.

**Objective:**

The aim of this paper was to evaluate the efficacy of enhanced self-management via a mobile app intervention on health-related quality of life, self-management, and HF readmissions.

**Methods:**

A single-center randomized controlled trial was performed. Participants older than 45 years and admitted for acute decompensated HF or recently discharged in the past 4 weeks were included. The intervention group (“app group”) used a mobile app, and the intervention prompted daily self-monitoring and promoted self-management. The control group (“no-app group”) received usual care. The primary outcome was the change in Minnesota Living with Heart Failure Questionnaire (MLHFQ) score from baseline to 6 and 12 weeks. Secondary outcomes were the Self-Care Heart Failure Index (SCHFI) questionnaire score and recurrent HF admissions.

**Results:**

A total of 83 participants were enrolled and completed all baseline assessments. Baseline characteristics were similar between the groups except for the prevalence of ischemic HF. The app group had a reduced MLHFQ at 6 weeks (mean 37.5, SD 3.5 vs mean 48.2, SD 3.7; *P*=.04) but not at 12 weeks (mean 44.2, SD 4 vs mean 45.9, SD 4; *P*=.78), compared to the no-app group. There was no effect of the app on the SCHFI at 6 or 12 weeks. The time to first HF readmission was not statistically different between the app group and the no-app group (app group 11/42, 26% vs no-app group 12/41, 29%; hazard ratio 0.89, 95% CI 0.39-2.02; *P*=.78) over 12 weeks.

**Conclusions:**

The adaptive mobile app intervention, which focused on promoting self-monitoring and self-management, improved the MLHFQ at 6 weeks but did not sustain its effects at 12 weeks. No effect was seen on HF self-management measured by self-report. Further research is needed to enhance engagement in the app for a longer period and to determine if the app can reduce HF readmissions in a larger study.

**Trial Registration:**

ClinicalTrials.gov NCT03149510; https://clinicaltrials.gov/ct2/show/NCT03149510

## Introduction

Despite major scientific advances, heart failure (HF) continues to be a common and costly condition; each year, over 1 million people are admitted to an inpatient setting for acute heart failure [1]. HF is the most common hospital discharge diagnosis among older adults in the United States, and one-fifth of HF patients are readmitted within 30 days of discharge [2].

Hospital readmissions are a substantial concern in HF and are directly linked to poor health-related quality of life (HRQOL) [3]. HF readmissions also result in significant, potentially avoidable costs to our already-strained health care system because hospitalizations account for nearly 70% of annual HF costs [1]. National attention has turned toward reducing 30-day readmissions for acute heart failure, partially because, in October 2012, the Centers for Medicare & Medicaid Services started to receive financial penalties for higher-than-expected rates of readmissions.

One of the most common causes of HF readmission—failure to recognize clinical worsening‌‌‌‌—is related to poor self-management [4,5]. HF care includes daily monitoring of weight and symptoms, taking medications as prescribed, adhering to a low-sodium diet, and assessing changes in symptoms related to self-monitoring [6]. Self-management is when a patient understands how to interpret self-monitoring to ultimately change their behaviors and improve symptoms. Increasing patients’ understanding of the link between self-monitoring and self-management is key to successful HF disease management interventions [7]. Several studies have shown that self-monitoring can enhance self-management and improve HRQOL in HF [8-10]. However, currently, there are few clinically effective HF self-management tools to support HF patients in managing their condition after they transition from the hospital back into the community. Thus, there is an urgent need for low-cost solutions to help patients recognize clinical worsening and reduce HF readmissions. This study’s objective was to evaluate the effectiveness of a mobile app intervention that enhances self-monitoring of HRQOL, self-management, and HF readmissions.

## Methods

### Study Design

This was a 12-week, prospective, single-center, open-label randomized controlled trial conducted at Michigan Medicine, the University of Michigan’s academic medical center. The trial was registered on ClinicalTrials.gov (NCT03149510) and approved by the University of Michigan’s Institutional Review Board. The participants were recruited from March 2017 to April 2019 by in-person recruitment from the inpatient adult hospital. The participants were randomized to the intervention (“app”) or control (“no app”) group in a 1:1 fashion using the Trial Randomize application created by the University of Michigan’s Consulting for Statistics, Computing and Analytics Research center. The randomization methodology uses a minimization approach to reduce covariate imbalances by using nonuniform assignment probabilities for the 2 groups [11]. All of the participants provided a written consent before being fully enrolled in the clinical trial.

### Study Participants

The participants were included if they were older than 45 years, had a left ventricular ejection fraction (EF) of ≤40% or >40% (with a left atrial size of >40 mm, brain natriuretic peptide of >200 pg/mL, or N-terminal pro–B-type natriuretic peptide of >800 pg/mL), and were currently admitted or recently discharged for acute on chronic decompensated HF. participants were excluded if they had unstable coronary syndromes within 8 weeks, primary valvular heart disease, constrictive pericardial disease, uncorrected thyroid disease, dialysis or creatinine of >4.0 mg/dL, active cancer, and pulmonary fibrosis. They were also excluded if they were a hospice candidate, if they were discharged to a setting other than home, or if they were requiring a chronic inotrope. The participants were not blinded due to the nature of the intervention. In May 2018, inclusion criteria were expanded to include HF with preserved ejection fraction, in addition to HF with reduced EF and those recently discharged to increase recruitment. Of the total 83 participants, 80 were enrolled during index hospitalization. The remaining 3 were enrolled within 4 weeks of discharge, at days 2, 4, and 28, respectively.

### Intervention

**Figure 1:**
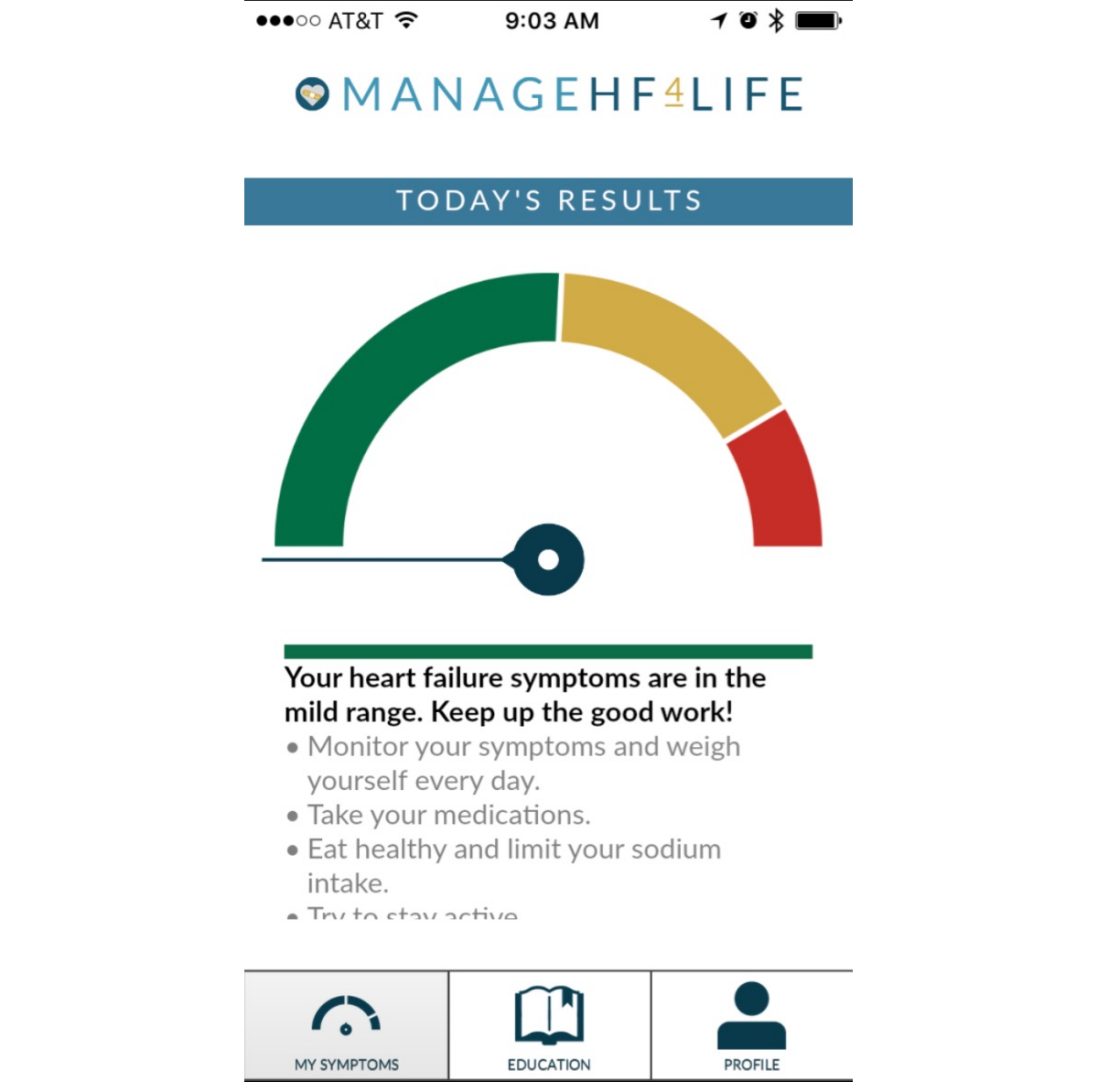
Example of a health status indicator in the ManageHF4Life mobile app.

The app group used a mobile app, ManageHF4Life, version 1 (The University of Michigan), along with a Fitbit (Fitbit Inc) physical activity monitor (Fitbit Charge 2) and scale (Fitbit Aria and Aria 2). Accurate self-monitoring, feedback, and self-efficacy are essential components for managing HF. The app prompted active daily self-monitoring, provided a health status indicator to promote self-management, and included standard education on HF. The daily prompt for active self-monitoring was carried out with a 9-AM push notification to complete an 8-question survey within the app. If the participants did not complete the survey by 12 PM, a reminder push notification was sent to them. The health status indicator was a stoplight (green, yellow, and red) and was generated from a rule-based model created by the investigators. The rule-based model was calculated from an equation based on the 8 survey questions and the difference between the daily weight and dry weight that was recorded in the app. The stoplight colors represented the participants’ health status: the green color represented stable status, while yellow and red represented a clinical worsening state. The text below the health status indicator changed based on the color, with recommendations on self-management. An example of a health status indicator screen is shown in Figure 1, and the full mobile app layout is presented in the supplement. All intervention participants were provided with a 30-minute educational session on how to use the app. The control group received usual care upon discharge from the hospital. At Michigan Medicine, all patients receive discharge education about heart failure, which includes self-monitoring, a 2-week follow-up appointment with an advanced practice provider, and periodic phone calls from a telehealth HF nurse. The Fitbit scale was used to record the daily weight, but the Fitbit physical activity monitor was not intentionally used as part of the self-monitoring intervention.

### Outcome Measures

The primary outcome was the change in Minnesota Living with Heart Failure Questionnaire (MLHFQ) from baseline to 6 and 12 weeks [12]. This tool consists of 21 questions regarding the patients' perception of the effects of HF on their daily lives. Secondary outcomes were the change in self-management and HF readmission over time. Self-management was measured using the Self-Care Heart Failure Index (SCHFI), version 6.2, which was the most current version available at trial initiation [13]. The SCHFI 6.2 contains 22 questions and has 3 subscales that determine the patient’s physiologic stability, response to symptoms, and ability to perform self-management. The questions in each subscale are standardized to a score of 0 to 100. Each subscale is added together to give the total SCHFI score. The SCHFI was collected at baseline, 6 weeks, and 12 weeks. Both the MLHFQ and the SCHFI were completed by participants using an automated online survey. All readmissions were reviewed in a blinded fashion for the potential to be an HF readmission. An unscheduled hospitalization was defined as an HF readmission if the primary diagnosis was HF and the length of stay either exceeded 24 hours or crossed a calendar day [14]. Outcome assessment was blinded to the randomization group. The study team contacted participants at 6 and 12 weeks to confirm the clinical outcomes and prompted the participants to complete any survey tasks. At the completion of the clinical trial, each participant in the app group received an online survey about the mobile app, which focused on its perceived usefulness and ease of use.

### Statistical Analysis

The primary outcome of the trial was the change in the MLHFQ between the app and no-app groups from baseline to 6 and 12 weeks, using a modified intention-to-treat approach. Repeated measures mixed models (SAS PROC MIXED, SAS Institute) were used to determine the change in MLHFQ score over 12 weeks between the 2 groups. The group indicator (app vs no app) served as the primary covariate, and least squares mean and standard error are reported for the continuous variables over time. Based on preliminary data [9], the MLHFQ score was expected to improve from 56 to 42 on average in the app group, with no change in the no-app group (SD 11.5). Based on these assumptions, 40 participants per group (N=80) with 20% dropout will have the power of more than 83% to detect the difference at the significance level of 0.05.

For baseline characteristics, continuous variables were compared using a *t* test, and categorical variables were compared using the chi-square or Fisher exact tests, where appropriate. Repeated measures mixed models were used to compare the change in the SCHFI over time between the app and no-app groups, and data are presented in least squares mean and standard error. Cox proportional hazards survival model was used to analyze time to HF readmission between the app and no-app groups.

## Results

### Baseline Characteristics

**Figure 2:**
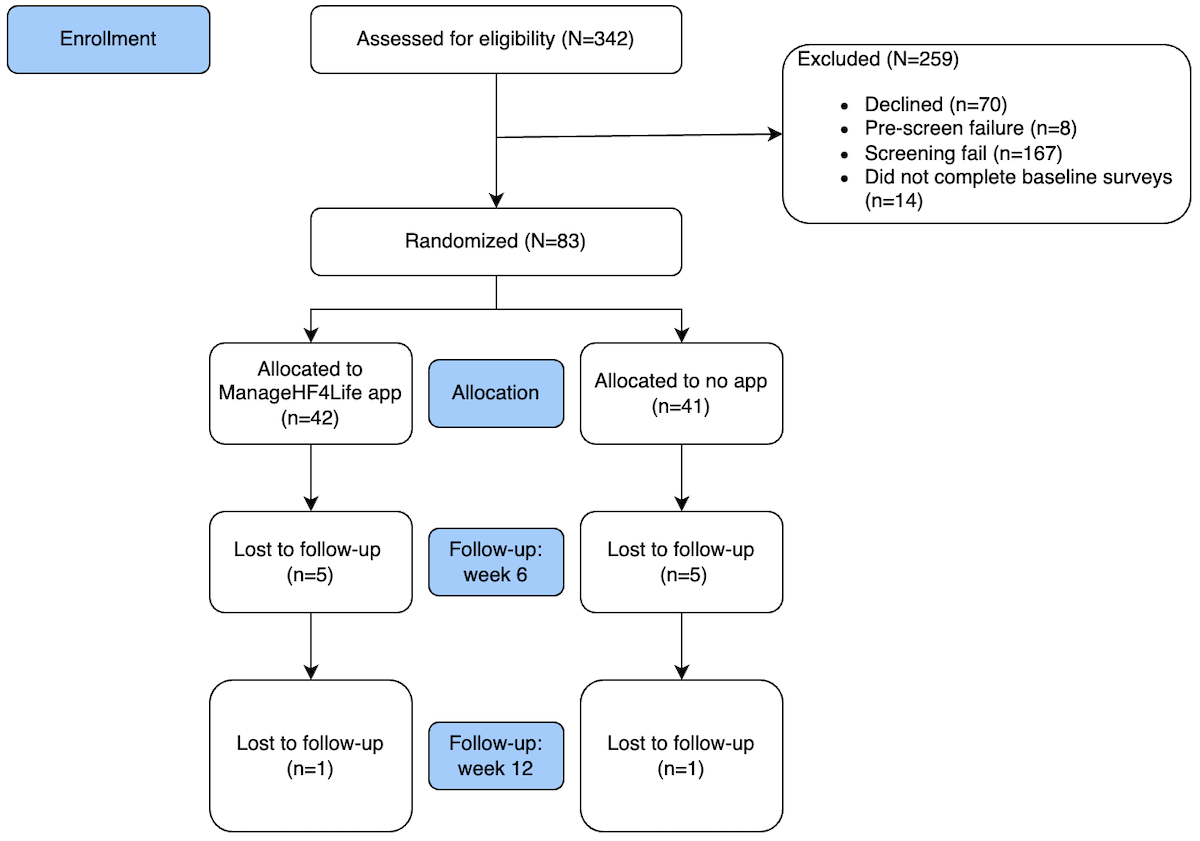
CONSORT (Consolidated Standards of Reporting Trials) flow diagram.

A total of 83 participants were enrolled and completed all baseline assessments. Baseline characteristics were similar between the groups except for the prevalence of ischemic HF. The participants were 60.2 (SD 9.2) years old in the app group and 62 (SD 9.2) years old in the no-app group (*P*=.38). The average EF was 37.2% in the app group and 38.2% in the no-app group (*P*=.73). Most of the participants were Caucasian: 81% (34/42) app vs 83% (34/41) no app (*P*=.56); most of the participants were also New York Heart Association (NYHA) class III: 55% (23/42) app vs 66% (27/41) no app (*P*=.41) at study enrollment. The median number of days during which the app group performed self-monitoring within the app was 63 (IQR 28-84) of the 84 days (75%). Figure 2 represents the CONSORT (Consolidated Standards of Reporting Trials) diagram for this clinical trial, and Table 1 demonstrates the baseline characteristics for the participants in both groups.

**Table 1:** Baseline demographics.

Variable		App (n=42)	No app (n=41)	*P* value	
Age (years), mean (SD)		60.2 (9)	62 (9)	.38	
Female, n (%)		14 (33)	15 (37)	.76	
**Race**					.56
	Caucasian, n (%)	34 (81)	34 (83)		
	African American, n (%)	7 (17)	6 (15)		
	Other, n (%)	1 (2)	1 (2)		
EF^a^ (%), mean (SD)		37.2 (20)	38.8 (19)	.73	
HFpEF^b^, n (%)		16 (38)	19 (46)	.45	
Ischemic HF^c^, n (%)		19 (45)	29 (71)	.02	
**NYHA^d^ class**					
	Class I, n (%)	1 (2)	0 (0)	.41	
	Class II, n (%)	10 (24)	5 (12)		
	Class III, n (%)	23 (55)	27 (66)		
	Class IV, n (%)	8 (19)	9 (22)		
Atrial fibrillation, n (%)		22 (52)	25 (61)	.43	
MI^e^, n (%)		10 (24)	18 (44)	.05	
DM^f^, n (%)		14 (33)	13 (32)	.87	
Moderate or severe renal disease, n (%)		1 (2)	2 (5)	.62	
Systolic BP^g^ (mm Hg), mean (SD)		121.1 (23)	119.1 (21)	.68	
Sodium (mmol/L), mean (SD)		138.3 (3)	137.8 (3)	.54	
Hemoglobin (g/dL), mean (SD)		12.4 (2)	11.9 (2)	.32	
ACEI^h^, ARB^i^, ARNI^j^, n (%)		28 (67)	20 (49)	.10	
Beta blocker, n (%)		37 (88)	35 (85)	.76	
MRA^k^, n (%)		18 (43)	16 (39)	.72	

^a^EF: ejection fraction.

^b^HFpEF: heart failure with preserved ejection fraction.

^c^HF: heart failure.

^d^NYHA: New York Heart Association.

^e^MI: myocardial infarction.

^f^DM: diabetes mellitus.

^g^BP: blood pressure.

^h^ACEI: angiotensin converting enzyme inhibitor.

^i^ARB: angiotensin receptor blocker.

^j^ARNI: angiotensin receptor and neprilysin inhibitor.

^k^MRA: mineralocorticoid receptor antagonist.

### MLHFQ Scores

**Figure 3:**
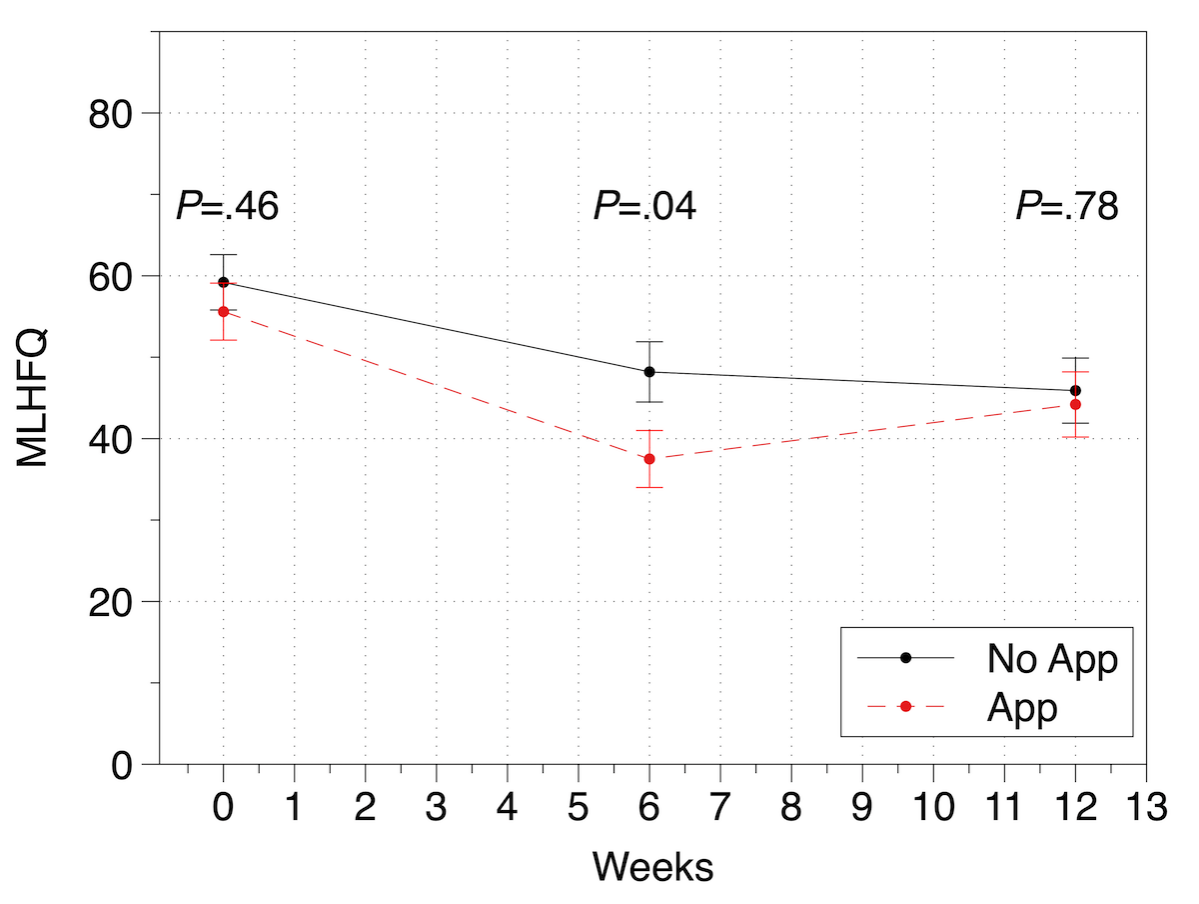
The change in Minnesota Living with Heart Failure Questionnaire (MLHFQ) score over time by group.

In the app group, the MLHFQ score changed from a baseline of 55.6 (SD 3.5) to 37.5 (SD 3.5) at 6 weeks and 44.2 (SD 4) at 12 weeks. The MLHFQ score in the no-app group changed from a baseline of 59.2 (SD 3.4) to 48.2 (SD 3.7) at 6 weeks and 45.9 (SD 4) at 12 weeks. The app group had a greater improvement in MLHFQ score at 6 weeks compared with the no-app group (*P*=.04), but not at 12 weeks (*P*=.78). Figure 3 demonstrates the change in MLHFQ total score over the course of the study between groups.

Among the emotional and physical subscales of the MLHFQ, the physical subscale showed similar results as the overall MLHFQ scale. MLHFQ physical scores changed from a baseline of 23.3 (SD 1.5) to 14.4 (SD 1.6) at 6 weeks and 17.8 (SD 1.9) at 12 weeks in the app group and a baseline of 24.4 (SD 1.5) to 20.4 (SD 1.7) at 6 weeks and 18.6 (SD 1.7) at 12 weeks in the no-app group. The app group had a greater improvement in the MLHFQ physical subscale at 6 weeks compared with the no-app group (*P*=.01), but not at 12 weeks (*P*=.78). MLHFQ emotional scores changed from a baseline of 12 (SD 1) to 8.7 (SD 1) at 6 weeks and 9.3 (SD 1.1) at 12 weeks in the app group and a baseline of 11.9 (SD 1.2) to 10 (SD 1.1) at 6 weeks and 10 (SD 1.2) at 12 weeks in the no-app group. The app group had similar changes in the MLHFQ emotional subscale at 6 weeks compared to the no-app group (*P*=.38) and at 12 weeks (*P*=.64).

### SCHFI Scores

The SCHFI total score changed from a baseline of 186.1 in the app group and 187.8 in the no-app group to 198.1 and 204.6, respectively, at 6 weeks (*P*=.40), and 196.9 and 206.1, at 12 weeks (*P*=.24). The maintenance, management, and confidence subscales of the SCHFI showed similar results. Table 2 demonstrates the change over time of the total SCHFI and 3 subscales in the app and no-app groups.

**Table 2:** The change in SCHFI and subscales over time by group.

Scores	Baseline				6 weeks				12 weeks		
	App	No app	*P* value	App		No app	*P* value	App		No app	*P* value
Total SCHFI, mean (SD)	186.1 (5)	187.8 (5)	.82	198.1 (5)		204.6 (5)	.40	196.9 (6)		206.1 (5)	.24
Maintenance, mean (SD)	66.6 (2)	70.4 (2)	.23	70.5 (2)		73.5 (2)	.37	69.9 (2)		74.6 (2)	.15
Management, mean (SD)	54.3 (2)	54.6 (2)	.94	55.7 (2)		60.4 (2)	.13	59.9 (2)		59 (2)	.78
Confidence, mean (SD)	64.5 (3)	62.9 (3)	.68	72.2 (3)		71.1 (3)	.79	67.7 (3)		72.6 (3)	.22

### Readmissions

**Figure 4:**
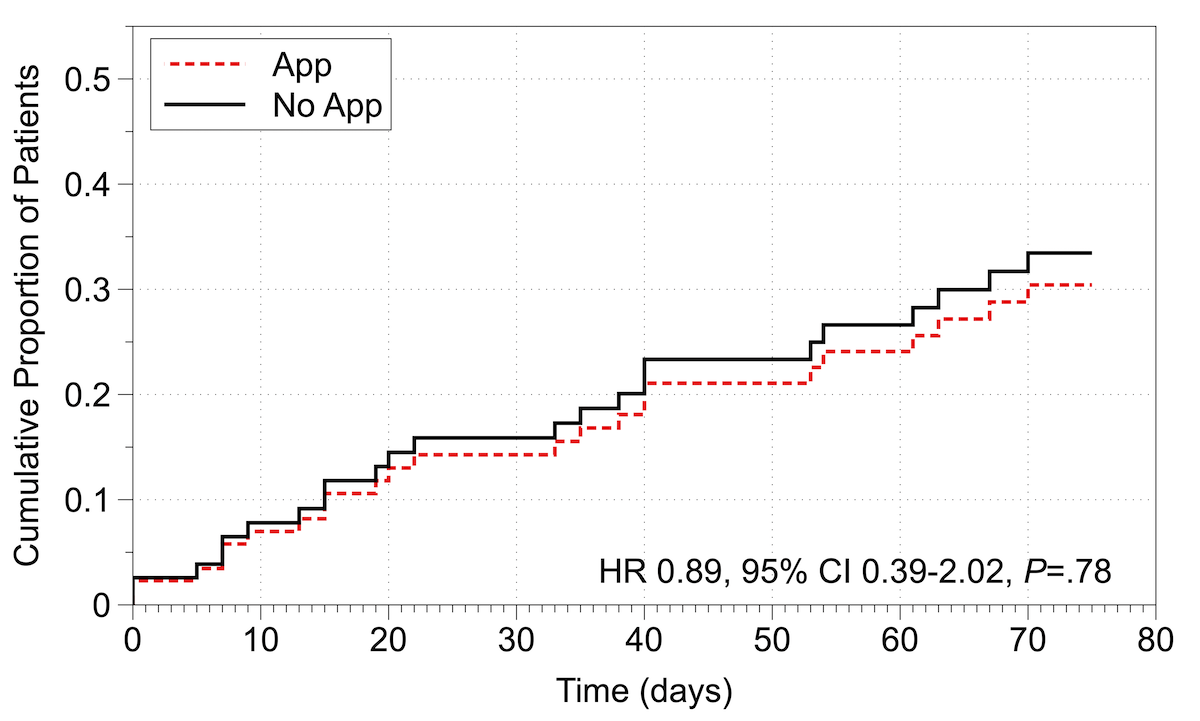
Time to first heart failure readmission by group; HR: heart rate.

Over the 12-week study, 26% (11/42) of the participants had an HF readmission in the app group compared with 29% (12/41) of the participants in the no-app group (hazard ratio 0.89, 95% CI 0.39-2.02; *P*=.78). There was no significant difference in HF readmission rates between the participants in the 2 groups. Figure 4 shows a plot of the time-to-event curves for the app and no-app groups. A total of 13 non–HF-related readmissions, 8 out of 42 (19%) in the app group and 5 out of 41 (12%) in the no-app group, occurred during the 12-week follow-up. One participant HF readmission event was followed by a death.

### Mobile App Survey

In total, 86% (36/42) of the app group participants completed a survey about the app, at the end of the trial. Of the 36 participants who completed the survey, 92% (n=33) agreed or strongly agreed that they found the app useful, 94% (n=34) agreed or strongly agreed that they used the information in the app in their daily life, 89% (n=32) agreed or strongly agreed that the information they received in the app was important to them, and 97% (n=35) agreed or strongly agreed that the app was easy to use. Only 3% (n=1) agreed that the app was confusing, and no one stated that the app was difficult to understand. Moreover, 92% (n=33) thought that most people would learn to use the mobile app quickly; 75% (n=27) agreed or strongly agreed that they learned a lot from the mobile app; and 58% (n=21) said the mobile app had new information that they were not aware of before.

## Discussion

### Principal Findings

In recent years, smartphones have changed the landscape of the US society with 81% of the population now owning a smartphone [15]. The widespread access to smartphones can be harnessed to dramatically change health care delivery. In this study, a mobile app that used a health status indicator to communicate a clinical worsening state showed a greater improvement in HRQOL at 6 weeks, but did not sustain effects at 12 weeks when compared to a control group. From the ESCAPE (Endovascular Treatment for Small Core and Anterior Circulation Proximal Occlusion with Emphasis on Minimizing CT to Recanalization Times) trial, a decrease in the MLHFQ total score of 20 points at 1 month after an HF discharge had a lower rate of death or hospitalization compared with a 10-point decrease at 1 month [16]. In our study, the ManageHF4Life intervention demonstrated an 18-point decrease from baseline to 6 weeks compared with an 11-point decrease in the control group. This shows that the 6-week findings are clinically meaningful and deserve future investigation. This effect was also primarily driven by improvements in the physical subscale of the MLHFQ as opposed to the emotional subscale. The physical and emotional subscales of the MLHFQ have been shown to characterize how HF is affecting a patient’s life. The physical subscale questions deal with the effects on the body, and the emotional subscale questions deal with the effects on the mind. The ManageHF4Life intervention is primarily targeted at the physical components of HF, so this finding aligns with the intended effects of the intervention.

Our study did not demonstrate an effect of the ManageHF4Life intervention on the secondary outcome of self-management, using the SCHFI score, compared to control. Self-management is affected directly or indirectly by depression, social support, eHealth literacy, and HF knowledge [16]. At baseline, the SCHFI total and subscale scores were higher in our study compared with those reported in the literature [13,17,18], which could have made it more difficult to demonstrate a change in self-management over time. SCHFI scores may have been higher at baseline and throughout our study, as many of the patients were followed in an advanced HF telemanagement program. This program is designed to provide clinical support and education to patients. The survey at the end of the study showed that 58% (21/36) of the participants said the ManageHF4Life intervention had new information that they were not aware of before. Increasing HF knowledge should increase self-management, but that was not the case in this study. In addition, the ManageHF4Life intervention did not improve the emotional subscale of the MLHFQ, which aligns with the depression and social support aspects of self-management. Future interventions should target a broader support for self-management, including depression, social support, and knowledge.

A recent integrative review found 18 publications that studied the effects of mobile apps for heart failure [19]. In those studies, the total sample size ranged from 7 to 165 participants, and 7 of them were randomized controlled trials. Similar to our app, 14 studies included apps that monitored self-management components (weight, blood pressure, and HF symptoms). One mobile app, HeartMapp (University of South Florida), was most similar to our study app [20]. HeartMapp used a built-in algorithm based on the NYHA classification presenting green, yellow, orange, and red zones. The app was studied in an 18-patient, 30-day randomized controlled pilot study of patients being enrolled at hospital discharge. The study aimed to determine if the mobile app, compared to control, improved HRQOL using the Kansas City Cardiomyopathy Questionnaire and self-management behaviors using the SCHFI. It is unclear which version of the SCHFI was used because the methods do not state the version, and the subscale numbers do not match those in the SCHFI, version 6.2. Although underpowered, this study demonstrated a significant improvement in the SCHFI self-management and self-confidence subscales in the mobile app group, compared with control. Kansas City Cardiomyopathy Questionnaire measurements did not change over time in this 30-day study.

Moreover, there are some main differences between our study and the HeartMapp study. Our app used a clinician-derived rule-based model that includes patient symptoms and the change in body weight, while HeartMapp used a NYHA-based algorithm. The HeartMapp study included an active control group that was given access to some of the features of the app, while, in our study, the control group received usual care and did not have access to the mobile app. It is not possible to compare the difference in the SCHFI results in our study and HeartMapp because the methods do not state the version, and subscale numbers do not match those in SCHFI, version 6.2.

### Limitations

While this is a randomized controlled trial, there are some limitations in the study. The study was open-label, so participants knew the group in which they were randomized. This could have led to a bias by participants in either group or provided undue influence on our results. The control group was “usual care” with no mobile app and did not include an attention control. Although it is common to use usual-care groups when studying mobile apps, attention control groups strengthen behavioral interventions [21]. Furthermore, the usual care in our center may be a more intensive care than some other centers in the country. Future studies of our mobile app should include a control group that receives the app, but not intervention components of interest. We gave all participants in the app group a wearable device and scale at the beginning of the study. This could have led to an intervention above and beyond the mobile app health status indicator. There are studies, however, that refute the idea that adding a wearable to an intervention improves outcomes more than the intervention alone [22]. In addition to these limitations, version 1 of our mobile app, ManageHF4Life, was very basic. It did not include contextual push notifications about self-management, adaptive content in the mobile app, or just-in-time dietary information when selecting foods [23]. Future research of the app will focus on these enhancements and other study designs to optimize the intervention and determine the effects on HF outcomes.

### Conclusions

The mobile app intervention improved MLHFQ at 6 weeks but did not sustain its effects at 12 weeks, compared to control. No effect was seen on self-management measured by self-report with the SCHFI. Further versions of the app should focus on technological enhancements, and future research is needed to determine if those future versions can reduce HF readmissions in a larger study.

## Appendix

Multimedia Appendix 1 CONSORT-eHEALTH checklist (V 1.6.2).

## Abbreviations

CONSORT: Consolidated Standards of Reporting Trials
EF: ejection fraction
ESCAPE: Endovascular Treatment for Small Core and Anterior Circulation Proximal Occlusion With Emphasis on Minimizing CT to Recanalization Times
HF: heart failure
HRQOL: health-related quality of life
MLHFQ: Minnesota Living with Heart Failure Questionnaire
NYHA: New York Heart Association
SCHFI: Self-Care Heart Failure Index
