# Supplementary material for: The Effects of the ManageHF4Life Mobile App on Patients With Chronic Heart Failure: Randomized Controlled Trial
Source: JMIR Mhealth Uhealth. 2021 Dec 7;9(12):e26185. doi: 10.2196/26185 (PMC8693200; doi:10.2196/26185)
Supplement: Multimedia Appendix 1 [file mhealth_v9i12e26185_app1.pdf]

CONSORT-EHEALTH Checklist V1.6.2 Report	Manuscript Number	26185
**(based on CONSORT-EHEALTH V1.6), available at [http://tinyurl.com/consort-ehealth-v1-6].**		
**Date completed**		
4/28/2021 15:30:56		
**by**		
Michael Dorsch		
The Effects of the ManageHF4Life Mobile Application on Patients With Chronic Heart Failure: Randomized Controlled Trial		
TITLE		
**1a-i) Identify the mode of delivery in the title**		
"The Effects of the ManageHF4Life Mobile Application on Patients With Chronic Heart Failure: Randomized Controlled Trial"		
**1a-ii) Non-web-based components or important co-interventions in title**		
**1a-iii) Primary condition or target group in the title**		
"The Effects of the ManageHF4Life Mobile Application on Patients With Chronic Heart Failure: Randomized Controlled Trial"		
ABSTRACT		
**1b-i) Key features/functionalities/components of the intervention and comparator in the METHODS section of the ABSTRACT**		
Objective: Evaluate the effectiveness of a mobile application intervention that enhances self-monitoring on health-related quality of life, self-management, and reduces HF readmissions.<br>Methods: A single center randomized controlled trial was performed. Participants greater than 45 years of age and admitted for acute decompensated HF or recently discharged in the past 4 weeks were included. The intervention group used a mobile application (App). The intervention prompted daily self-monitoring and promoted self-management. The control group (No App) received usual care. The primary outcome was the change in Minnesota Living with Heart Failure Questionnaire (MLHFQ) from baseline to 6 and 12 weeks. Secondary outcomes were the Self-Care Heart Failure Index (SCHFI) questionnaire and recurrent HF admissions.		
**1b-ii) Level of human involvement in the METHODS section of the ABSTRACT**		
**1b-iii) Open vs. closed, web-based (self-assessment) vs. face-to-face assessments in the METHODS section of the ABSTRACT**		
**1b-iv) RESULTS section in abstract must contain use data**		
**1b-v) CONCLUSIONS/DISCUSSION in abstract for negative trials**		
INTRODUCTION		
**2a-i) Problem and the type of system/solution**		
"The App group used a mobile application, ManageHF4Life version 1, along with a Fitbit physical activity monitor (Fitbit Charge 2) and scale (Fitbit Aria and Aria 2). The mobile application was created from the theory of self-regulation and developed using user centered design. Accurate self-monitoring, feedback and self-efficacy are essential components of the self-regulation cycle and are critical for managing HF. The application prompted active daily self-monitoring, provided a health status indicator to promote self-management, and included standard education on HF. The daily prompt for active self-monitoring was done with a 9:00 a.m. push notification to complete an 8-question survey within the application. If they did not complete the survey by 12:00 p.m., a reminder push notification was sent to the user. The health status indicator was a stop light (green, yellow, and red) and was generated from a rule-based model created by the investigators. The rule-based model was calculated from an equation based on the eight survey questions and the difference between the daily weight and dry weight that was recorded in the application. The stop light colors represented the participants' health status. The green color represented stable status. Yellow and red represented a clinical worsening state. The text below the health status indicator changed based on the color with recommendations on self-management. An example health status indicator screen is shown in figure 1 and the full mobile application layout is presented in the supplement. All intervention participants were provided a 30-minute educational session on how to use the application. The control group received usual care upon discharge from the hospital. At Michigan Medicine, usual care is a two week follow up appointment with an advanced practice provider and periodic phone calls from a telehealth HF nurse."		
**2a-ii) Scientific background, rationale: What is known about the (type of) system**		
"One of the most common causes of HF readmission – failure to recognize clinical worsening – is related to poor self-management.[4,5] HF care includes daily monitoring of weight and symptoms, taking medications as prescribed, adhering to a low sodium diet, and assessing changes in symptoms related to self-monitoring. Self-management is when a patient understands how to interpret self-monitoring to ultimately change their behaviors and improve symptoms. Increasing patient understanding of the link between self-monitoring and self-management is the key to successful HF disease management interventions.[6] Several studies have shown that self-monitoring can enhance self-management and improve HRQOL in HF.[7-9] However, currently, there are few clinically effective HF self-management tools to support HF patients in managing their condition after they transition from the hospital back into the community. Thus, there is an urgent need for low-cost solutions to help patients recognize clinical worsening and reduce HF readmissions. This study's objective was to evaluate the effectiveness of a mobile application intervention that enhances self-monitoring on HRQOL, self-management and HF readmissions."		
**Does your paper address CONSORT subitem 2b?**		
"This study's objective was to evaluate the effectiveness of a mobile application intervention that enhances self-monitoring on HRQOL, self-management and HF readmissions."		
METHODS		
**3a) CONSORT: Description of trial design (such as parallel, factorial) including allocation ratio**		
"This was a 12-week prospective, single-center, open-label, randomized controlled trial conducted at Michigan Medicine, the University of Michigan academic medical center. The trial was registered on clinicaltrials.gov (NCT03149510) and approved by the University of Michigan Institutional Review Board. Participants were recruited from March 2017 to April 2019 by in-person recruitment from the inpatient adult hospital. Participants were randomized to the intervention (App) or control (No App) group in a 1:1 fashion using the Trial Randomize (https://trial-randomize.appspot.com/) application created by the University of Michigan Consulting for Statistics, Computing and Analytics Research (CSCAR). The randomization methodology uses the minimization approach to reduce covariate imbalances by using non-uniform assignment probabilities for the two groups.[10] All participants provided a written consent before being fully enrolled in the clinical trial."		
**3b) CONSORT: Important changes to methods after trial commencement (such as eligibility criteria), with reasons**		
"This was a 12-week prospective, single-center, open-label, randomized controlled trial conducted at Michigan Medicine, the University of Michigan academic medical center. The trial was registered on clinicaltrials.gov (NCT03149510) and approved by the University of Michigan Institutional Review Board. Participants were recruited from March 2017 to April 2019 by in-person recruitment from the inpatient adult hospital. Participants were randomized to the intervention (App) or control (No App) group in a 1:1 fashion using the Trial Randomize (https://trial-randomize.appspot.com/) application created by the University of Michigan Consulting for Statistics, Computing and Analytics Research (CSCAR). The randomization methodology uses the minimization approach to reduce covariate imbalances by using non-uniform assignment probabilities for the two groups.[10] All participants provided a written consent before being fully enrolled in the clinical trial. "		
**3b-i) Bug fixes, Downtimes, Content Changes**		
**4a) CONSORT: Eligibility criteria for participants**		
"Participants were included if they were greater than 45 years of age, had a left ventricular ejection fraction (LVEF) </= 40% or a LVEF >40% (with LA size >40 mm or BNP > 200 pg/ml or NT-proBNP > 800 pg/ml) and were currently admitted or recently discharged for acute on chronic decompensated HF. Participants were excluded for any of the following: unstable coronary syndromes within 8 weeks, primary valvular heart disease, constrictive pericardial disease, uncorrected thyroid disease, dialysis or creatinine >4.0 mg/dL, a hospice candidate, active cancer, pulmonary fibrosis, discharged to a setting other than home, or required a chronic inotrope. Participants were not blinded due to the nature of the intervention. In May 2018, inclusion criteria were expanded to include HF with preserved ejection fraction in addition to HF with reduced ejection fraction and those recently discharged to increase recruitment. Eighty participants were enrolled during the index hospitalization. The remaining three of the eighty-three participants were enrolled within four weeks of discharge, at day 2, 4, and 28, respectively."		
**4a-i) Computer / Internet literacy**		
**4a-ii) Open vs. closed, web-based vs. face-to-face assessments:**		
"Participants were included if they were greater than 45 years of age, had a left ventricular ejection fraction (LVEF) </= 40% or a LVEF >40% (with LA size >40 mm or BNP > 200 pg/ml or NT-proBNP > 800 pg/ml) and were currently admitted or recently discharged for acute on chronic decompensated HF. Participants were excluded for any of the following: unstable coronary syndromes within 8 weeks, primary valvular heart disease, constrictive pericardial disease, uncorrected thyroid disease, dialysis or creatinine >4.0 mg/dL, a hospice candidate, active cancer, pulmonary fibrosis, discharged to a setting other than home, or required a chronic inotrope. Participants were not blinded due to the nature of the intervention. In May 2018, inclusion criteria were expanded to include HF with preserved ejection fraction in addition to HF with reduced ejection fraction and those recently discharged to increase recruitment. Eighty participants were enrolled during the index hospitalization. The remaining three of the eighty-three participants were enrolled within four weeks of discharge, at day 2, 4, and 28, respectively."		

**4a-iii) Information giving during recruitment**		
**4b) CONSORT: Settings and locations where the data were collected**		
"The primary outcome was the change in Minnesota Living with HF Questionnaire (MLHFQ) from baseline to 6 and 12 weeks.[11] This tool consists of 21 questions regarding patients' perception of the effects of HF on their daily lives. Secondary outcomes were the change in self-management and HF readmission over time. Self-management was measured using the Self-Care Heart Failure Index (SCHFI) version 6.2, which was the most current version available at trial initiation.[12] The SCHFI 6.2 contains 22 questions and has 3 subscales that determine the patient's physiologic stability, response to symptoms and ability to perform self-management. The questions in each subscale are standardized to a score of 0 to 100. Each subscale is added together to give the total SCHFI score. The SCHFI was collected at baseline, 6 and 12 weeks. Both the MLHFQ and SCHFI were complete by participants using an automated online survey. "		
**4b-i) Report if outcomes were (self-)assessed through online questionnaires**		
"The primary outcome was the change in Minnesota Living with HF Questionnaire (MLHFQ) from baseline to 6 and 12 weeks.[11] This tool consists of 21 questions regarding patients' perception of the effects of HF on their daily lives. Secondary outcomes were the change in self-management and HF readmission over time. Self-management was measured using the Self-Care Heart Failure Index (SCHFI) version 6.2, which was the most current version available at trial initiation.[12] The SCHFI 6.2 contains 22 questions and has 3 subscales that determine the patient's physiologic stability, response to symptoms and ability to perform self-management. The questions in each subscale are standardized to a score of 0 to 100. Each subscale is added together to give the total SCHFI score. The SCHFI was collected at baseline, 6 and 12 weeks. Both the MLHFQ and SCHFI were complete by participants using an automated online survey. "		
**4b-ii) Report how institutional affiliations are displayed**		
**5) CONSORT: Describe the interventions for each group with sufficient details to allow replication, including how and when they were actually administered**		
**5-i) Mention names, credential, affiliations of the developers, sponsors, and owners**		
**5-ii) Describe the history/development process**		
**5-iii) Revisions and updating**		
**5-iv) Quality assurance methods**		
**5-v) Ensure replicability by publishing the source code, and/or providing screenshots/screen-capture video, and/or providing flowcharts of the algorithms used**		
**5-vi) Digital preservation**		
**5-vii) Access**		
Participants were provided access with a user generated username and password.		
**5-viii) Mode of delivery, features/functionalities/components of the intervention and comparator, and the theoretical framework**		
"The App group used a mobile application, ManageHF4Life version 1, along with a Fitbit physical activity monitor (Fitbit Charge 2) and scale (Fitbit Aria and Aria 2). The mobile application was created from the theory of self-regulation and developed using user centered design. Accurate self-monitoring, feedback and self-efficacy are essential components of the self-regulation cycle and are critical for managing HF. The application prompted active daily self-monitoring, provided a health status indicator to promote self-management, and included standard education on HF. The daily prompt for active self-monitoring was done with a 9:00 a.m. push notification to complete an 8-question survey within the application. If they did not complete the survey by 12:00 p.m., a reminder push notification was sent to the user. The health status indicator was a stop light (green, yellow, and red) and was generated from a rule-based model created by the investigators. The rule-based model was calculated from an equation based on the eight survey questions and the difference between the daily weight and dry weight that was recorded in the application. The stop light colors represented the participants' health status. The green color represented stable status. Yellow and red represented a clinical worsening state. The text below the health status indicator changed based on the color with recommendations on self-management. An example health status indicator screen is shown in figure 1 and the full mobile application layout is presented in the supplement. All intervention participants were provided a 30-minute educational session on how to use the application. The control group received usual care upon discharge from the hospital. At Michigan Medicine, usual care is a two week follow up appointment with an advanced practice provider and periodic phone calls from a telehealth HF nurse."		
**5-ix) Describe use parameters**		
**5-x) Clarify the level of human involvement**		
**5-xi) Report any prompts/reminders used**		
"The App group used a mobile application, ManageHF4Life version 1, along with a Fitbit physical activity monitor (Fitbit Charge 2) and scale (Fitbit Aria and Aria 2). The mobile application was created from the theory of self-regulation and developed using user centered design. Accurate self-monitoring, feedback and self-efficacy are essential components of the self-regulation cycle and are critical for managing HF. The application prompted active daily self-monitoring, provided a health status indicator to promote self-management, and included standard education on HF. The daily prompt for active self-monitoring was done with a 9:00 a.m. push notification to complete an 8-question survey within the application. If they did not complete the survey by 12:00 p.m., a reminder push notification was sent to the user. The health status indicator was a stop light (green, yellow, and red) and was generated from a rule-based model created by the investigators. The rule-based model was calculated from an equation based on the eight survey questions and the difference between the daily weight and dry weight that was recorded in the application. The stop light colors represented the participants' health status. The green color represented stable status. Yellow and red represented a clinical worsening state. The text below the health status indicator changed based on the color with recommendations on self-management. An example health status indicator screen is shown in figure 1 and the full mobile application layout is presented in the supplement. All intervention participants were provided a 30-minute educational session on how to use the application. The control group received usual care upon discharge from the hospital. At Michigan Medicine, usual care is a two week follow up appointment with an advanced practice provider and periodic phone calls from a telehealth HF nurse."		
**5-xii) Describe any co-interventions (incl. training/support)**		
"The App group used a mobile application, ManageHF4Life version 1, along with a Fitbit physical activity monitor (Fitbit Charge 2) and scale (Fitbit Aria and Aria 2). The mobile application was created from the theory of self-regulation and developed using user centered design. Accurate self-monitoring, feedback and self-efficacy are essential components of the self-regulation cycle and are critical for managing HF. The application prompted active daily self-monitoring, provided a health status indicator to promote self-management, and included standard education on HF. The daily prompt for active self-monitoring was done with a 9:00 a.m. push notification to complete an 8-question survey within the application. If they did not complete the survey by 12:00 p.m., a reminder push notification was sent to the user. The health status indicator was a stop light (green, yellow, and red) and was generated from a rule-based model created by the investigators. The rule-based model was calculated from an equation based on the eight survey questions and the difference between the daily weight and dry weight that was recorded in the application. The stop light colors represented the participants' health status. The green color represented stable status. Yellow and red represented a clinical worsening state. The text below the health status indicator changed based on the color with recommendations on self-management. An example health status indicator screen is shown in figure 1 and the full mobile application layout is presented in the supplement. All intervention participants were provided a 30-minute educational session on how to use the application. The control group received usual care upon discharge from the hospital. At Michigan Medicine, usual care is a two week follow up appointment with an advanced practice provider and periodic phone calls from a telehealth HF nurse."		
**6a) CONSORT: Completely defined pre-specified primary and secondary outcome measures, including how and when they were assessed**		
"The primary outcome was the change in Minnesota Living with HF Questionnaire (MLHFQ) from baseline to 6 and 12 weeks.[11] This tool consists of 21 questions regarding patients' perception of the effects of HF on their daily lives. Secondary outcomes were the change in self-management and HF readmission over time. Self-management was measured using the Self-Care Heart Failure Index (SCHFI) version 6.2, which was the most current version available at trial initiation.[12] The SCHFI 6.2 contains 22 questions and has 3 subscales that determine the patient's physiologic stability, response to symptoms and ability to perform self-management. The questions in each subscale are standardized to a score of 0 to 100. Each subscale is added together to give the total SCHFI score. The SCHFI was collected at baseline, 6 and 12 weeks. Both the MLHFQ and SCHFI were complete by participants using an automated online survey. All readmissions were reviewed in a blinded fashion for the potential to be a HF readmission. An unscheduled hospitalization was defined as a HF readmission if the primary diagnosis was HF and the length of stay either exceeded 24 hours or crosses a calendar day.[13] Outcome assessment was done blinded to randomization group. The study team contacted participants at 6 and 12 weeks to confirm the clinical outcomes and prompted participants to complete any survey tasks. At the completion of the clinical trial, each participant in the App group received an online survey about the mobile application. The survey focused on the perceived usefulness and ease of use for the mobile application."		
**6a-i) Online questionnaires: describe if they were validated for online use and apply CHERRIES items to describe how the questionnaires were designed/deployed**		
**6a-ii) Describe whether and how "use" (including intensity of use/dosage) was defined/measured/monitored**		
**6a-iii) Describe whether, how, and when qualitative feedback from participants was obtained**		
**6b) CONSORT: Any changes to trial outcomes after the trial commenced, with reasons**		

"The primary outcome was the change in Minnesota Living with HF Questionnaire (MLHFQ) from baseline to 6 and 12 weeks.[11] This tool consists of 21 questions regarding patients' perception of the effects of HF on their daily lives. Secondary outcomes were the change in self-management and HF readmission over time. Self-management was measured using the Self-Care Heart Failure Index (SCHFI) version 6.2, which was the most current version available at trial initiation.[12] The SCHFI 6.2 contains 22 questions and has 3 subscales that determine the patient's physiologic stability, response to symptoms and ability to perform self-management. The questions in each subscale are standardized to a score of 0 to 100. Each subscale is added together to give the total SCHFI score. The SCHFI was collected at baseline, 6 and 12 weeks. Both the MLHFQ and SCHFI were complete by participants using an automated online survey. "

7a) CONSORT: How sample size was determined

7a-i) Describe whether and how expected attrition was taken into account when calculating the sample size

7b) CONSORT: When applicable, explanation of any interim analyses and stopping guidelines

"The primary outcome was the change in Minnesota Living with HF Questionnaire (MLHFQ) from baseline to 6 and 12 weeks.[11] This tool consists of 21 questions regarding patients' perception of the effects of HF on their daily lives. Secondary outcomes were the change in self-management and HF readmission over time. Self-management was measured using the Self-Care Heart Failure Index (SCHFI) version 6.2, which was the most current version available at trial initiation.[12] The SCHFI 6.2 contains 22 questions and has 3 subscales that determine the patient's physiologic stability, response to symptoms and ability to perform self-management. The questions in each subscale are standardized to a score of 0 to 100. Each subscale is added together to give the total SCHFI score. The SCHFI was collected at baseline, 6 and 12 weeks. Both the MLHFQ and SCHFI were complete by participants using an automated online survey. All readmissions were reviewed in a blinded fashion for the potential to be a HF readmission. An unscheduled hospitalization was defined as a HF readmission if the primary diagnosis was HF and the length of stay either exceeded 24 hours or crosses a calendar day.[13] Outcome assessment was done blinded to randomization group. The study team contacted participants at 6 and 12 weeks to confirm the clinical outcomes and prompted participants to complete any survey tasks. At the completion of the clinical trial, each participant in the App group received an online survey about the mobile application. The survey focused on the perceived usefulness and ease of use for the mobile application."

8a) CONSORT: Method used to generate the random allocation sequence

"Participants were randomized to the intervention (App) or control (No App) group in a 1:1 fashion using the Trial Randomize (https://trial-randomize. appspot.com/) application created by the University of Michigan Consulting for Statistics, Computing and Analytics Research (CSCAR). The randomization methodology uses the minimization approach to reduce covariate imbalances by using non-uniform assignment probabilities for the two groups.[10]"

8b) CONSORT: Type of randomisation; details of any restriction (such as blocking and block size)

"Participants were randomized to the intervention (App) or control (No App) group in a 1:1 fashion using the Trial Randomize (https://trial-randomize. appspot.com/) application created by the University of Michigan Consulting for Statistics, Computing and Analytics Research (CSCAR). The randomization methodology uses the minimization approach to reduce covariate imbalances by using non-uniform assignment probabilities for the two groups.[10]"

9) CONSORT: Mechanism used to implement the random allocation sequence (such as sequentially numbered containers), describing any steps taken to conceal the sequence until interventions were assigned

This was an open label study so concealment was not an issue.

10) CONSORT: Who generated the random allocation sequence, who enrolled participants, and who assigned participants to interventions

"Participants were randomized to the intervention (App) or control (No App) group in a 1:1 fashion using the Trial Randomize (https://trial-randomize. appspot.com/) application created by the University of Michigan Consulting for Statistics, Computing and Analytics Research (CSCAR). The randomization methodology uses the minimization approach to reduce covariate imbalances by using non-uniform assignment probabilities for the two groups.[10]"

11a) CONSORT: Blinding - If done, who was blinded after assignment to interventions (for example, participants, care providers, those assessing outcomes) and how

11a-i) Specify who was blinded, and who wasn't

Blinding was not performed in this trial.

11a-ii) Discuss e.g., whether participants knew which intervention was the "intervention of interest" and which one was the "comparator"

11b) CONSORT: If relevant, description of the similarity of interventions

"The App group used a mobile application, ManageHF4Life version 1, along with a Fitbit physical activity monitor (Fitbit Charge 2) and scale (Fitbit Aria and Aria 2). The mobile application was created from the theory of self-regulation and developed using user centered design. Accurate self-monitoring, feedback and self-efficacy are essential components of the self-regulation cycle and are critical for managing HF. The application prompted active daily self-monitoring, provided a health status indicator to promote self-management, and included standard education on HF. The daily prompt for active self-monitoring was done with a 9:00 a.m. push notification to complete an 8-question survey within the application. If they did not complete the survey by 12:00 p.m., a reminder push notification was sent to the user. The health status indicator was a stop light (green, yellow, and red) and was generated from a rule-based model created by the investigators. The rule-based model was calculated from an equation based on the eight survey questions and the difference between the daily weight and dry weight that was recorded in the application. The stop light colors represented the participants' health status. The green color represented stable status. Yellow and red represented a clinical worsening state. The text below the health status indicator changed based on the color with recommendations on self-management. An example health status indicator screen is shown in figure 1 and the full mobile application layout is presented in the supplement. All intervention participants were provided a 30-minute educational session on how to use the application. The control group received usual care upon discharge from the hospital. At Michigan Medicine, usual care is a two week follow up appointment with an advanced practice provider and periodic phone calls from a telehealth HF nurse."

12a) CONSORT: Statistical methods used to compare groups for primary and secondary outcomes

"The primary outcome of the trial was the change in MLHFQ between the App and No App groups from baseline to 6 and 12 weeks using modified intention-to-treat. Repeated measures mixed models (SAS proc mixed) was used to determine the change in MLHFQ score over 12-weeks between the two groups. The group indicator (App vs. No App) served as the primary covariate and least squares mean and standard error are reported for the continuous variables over time. Based on preliminary data[9], the MLHFQ score was expected to improve from 56 to 42 on average in the application group with no change in the usual care group (standard deviation of 11.5). Based on these assumptions, 40 participants per group (total N=80) with 20% dropout will have the power of more than 83% to detect the difference at the significance level of 0.05.

Continuous variables were compared using a t-test and categorical variables were compared using chi-square or Fisher's exact test where appropriate. Repeated measures mixed models was used to compare the change in SCHFI over time and data are presented in least squares mean and standard error. Cox proportional hazards survival model was used to analyze time to HF readmission. "

12a-i) Imputation techniques to deal with attrition / missing values

Imputation was not performed in the data analysis for this trial.

12b) CONSORT: Methods for additional analyses, such as subgroup analyses and adjusted analyses

Subgroup analyses and adjusted analyses were not performed in the data analysis for this trial.

RESULTS

13a) CONSORT: For each group, the numbers of participants who were randomly assigned, received intended treatment, and were analysed for the primary outcome

"Eighty-three participants were enrolled and completed all baseline assessments. Baseline characteristics were similar between groups except for the prevalence of ischemic HF. Participants were 60.2 years of age in the App group and 62 years of age in the No App group (P=0.379). The average ejection fraction (EF) was 37.2% in the App group and 38.2% in the No App group (P=0.725). Most of the participants were Caucasian (81% App vs. 83% No App, P=0.559) and NYHA class III (55% App vs. 66% No App, P=0.409) at study enrollment. The median number of days the App group performed self-monitoring within the application was 63 [IQR 28, 84] of the 84 days (74%). Table 1 demonstrates the baseline characteristics for the participants in both groups. Figure 2 represents the consort diagram for this clinical trial."

13b) CONSORT: For each group, losses and exclusions after randomisation, together with reasons

"Eighty-three participants were enrolled and completed all baseline assessments. Baseline characteristics were similar between groups except for the prevalence of ischemic HF. Participants were 60.2 years of age in the App group and 62 years of age in the No App group (P=0.379). The average ejection fraction (EF) was 37.2% in the App group and 38.2% in the No App group (P=0.725). Most of the participants were Caucasian (81% App vs. 83% No App, P=0.559) and NYHA class III (55% App vs. 66% No App, P=0.409) at study enrollment. The median number of days the App group performed self-monitoring within the application was 63 [IQR 28, 84] of the 84 days (74%). Table 1 demonstrates the baseline characteristics for the participants in both groups. Figure 2 represents the consort diagram for this clinical trial."

13b-i) Attrition diagram

14a) CONSORT: Dates defining the periods of recruitment and follow-up

"This was a 12-week prospective, single-center, open-label, randomized controlled trial conducted at Michigan Medicine, the University of Michigan academic medical center. The trial was registered on clinicaltrials.gov (NCT03149510) and approved by the University of Michigan Institutional Review Board. Participants were recruited from March 2017 to April 2019 by in-person recruitment from the inpatient adult hospital. "

14a-i) Indicate if critical "secular events" fell into the study period

14b) CONSORT: Why the trial ended or was stopped (early)

The trial was not stopped or ended early.

15) CONSORT: A table showing baseline demographic and clinical characteristics for each group

"Eighty-three participants were enrolled and completed all baseline assessments. Baseline characteristics were similar between groups except for the prevalence of ischemic HF. Participants were 60.2 years of age in the App group and 62 years of age in the No App group (P=0.379). The average ejection fraction (EF) was 37.2% in the App group and 38.2% in the No App group (P=0.725). Most of the participants were Caucasian (81% App vs. 83% No App, P=0.559) and NYHA class III (55% App vs. 66% No App, P=0.409) at study enrollment. The median number of days the App group performed self-monitoring within the application was 63 [IQR 28, 84] of the 84 days (74%). Table 1 demonstrates the baseline characteristics for the participants in both groups. Figure 2 represents the consort diagram for this clinical trial."		
**15-i) Report demographics associated with digital divide issues**		
"Eighty-three participants were enrolled and completed all baseline assessments. Baseline characteristics were similar between groups except for the prevalence of ischemic HF. Participants were 60.2 years of age in the App group and 62 years of age in the No App group (P=0.379). The average ejection fraction (EF) was 37.2% in the App group and 38.2% in the No App group (P=0.725). Most of the participants were Caucasian (81% App vs. 83% No App, P=0.559) and NYHA class III (55% App vs. 66% No App, P=0.409) at study enrollment. The median number of days the App group performed self-monitoring within the application was 63 [IQR 28, 84] of the 84 days (74%). Table 1 demonstrates the baseline characteristics for the participants in both groups. Figure 2 represents the consort diagram for this clinical trial."		
**16a) CONSORT: For each group, number of participants (denominator) included in each analysis and whether the analysis was by original assigned groups**		
**16-i) Report multiple "denominators" and provide definitions**		
The numbers are clearly defined for the results section.		
**16-ii) Primary analysis should be intent-to-treat**		
**17a) CONSORT: For each primary and secondary outcome, results for each group, and the estimated effect size and its precision (such as 95% confidence interval)**		
Yes, this was provided in the results section of the manuscript.		
**17a-i) Presentation of process outcomes such as metrics of use and intensity of use**		
**17b) CONSORT: For binary outcomes, presentation of both absolute and relative effect sizes is recommended**		
Yes, this was provided in the results section of the manuscript.		
**18) CONSORT: Results of any other analyses performed, including subgroup analyses and adjusted analyses, distinguishing pre-specified from exploratory**		
Yes, this was provided in the results section of the manuscript.		
**18-i) Subgroup analysis of comparing only users**		
**19) CONSORT: All important harms or unintended effects in each group**		
Yes, this was provided in the results section of the manuscript.		
**19-i) Include privacy breaches, technical problems**		
**19-ii) Include qualitative feedback from participants or observations from staff/researchers**		
DISCUSSION		
**20) CONSORT: Trial limitations, addressing sources of potential bias, imprecision, multiplicity of analyses**		
**20-i) Typical limitations in ehealth trials**		
"While this is a randomized controlled trial, there are some limitations in the study. The study was open-label so participants knew the group in which they were randomized. This could have led to a bias by participants in either group or provided undue influence on our results. The control group was usual care with no mobile application and did not include an attention control. Although it is common to use usual care groups when studying mobile applications, attention control groups strengthen behavioral interventions.[20] Furthermore, the usual care in our center may be a more intensive care than some other centers in the country. Future studies of our mobile application should include a control group that receives the application, but not intervention components of interest. We gave all participants in the App group a wearable device and scale at the beginning of the study. This could have led to an intervention above and beyond the mobile application health status indicator. There are studies, however, that refute the idea that adding a wearable to an intervention improves outcomes more than the intervention alone.[21] In addition to these limitations, version 1 of our mobile application, ManageHF4Life, was very basic. It did not include contextual push notifications about self-management, adaptive content in the mobile application or just-in-time dietary information when selecting foods.[22] Future research of the application will focus on these enhancements and other study designs to optimize the intervention and determine the effects on HF outcomes."		
**21) CONSORT: Generalisability (external validity, applicability) of the trial findings**		
**21-i) Generalizability to other populations**		
**21-ii) Discuss if there were elements in the RCT that would be different in a routine application setting**		
**22) CONSORT: Interpretation consistent with results, balancing benefits and harms, and considering other relevant evidence**		
**22-i) Restate study questions and summarize the answers suggested by the data, starting with primary outcomes and process outcomes (use)**		
"In this study, a mobile application that used a health status indicator to communicate a clinical worsening state showed a greater improvement in HRQOL at 6 weeks but did not sustain effects at 12 weeks when compared to a control group. From the ESCAPE trial, a decrease in the MLHFQ total score of 20 points at one month after a HF discharge had a lower rate of death or hospitalization compared to a 10-point decrease at one month.[15] In our study, the ManageHF4Life intervention demonstrated an 18-point decrease from baseline to 6 weeks compared to a 11-point decrease in the control group. This shows that the 6-week findings are clinically meaningful and deserve future investigation. This effect was also primarily driven by improvements in the physical subscale of the MLHFQ as opposed to the emotional subscale. The physical and emotional subscales of the MLHFQ have been shown to characterize how HF is affecting a patient's life. The physical subscale questions deal with the effects on the body and the emotional subscale questions deal with the effects on the mind. The ManageHF4Life intervention is primarily targeted at the physical components of HF, so this finding aligns with the intended effects of the intervention."		
**22-ii) Highlight unanswered new questions, suggest future research**		
Other information		
**23) CONSORT: Registration number and name of trial registry**		
ClinicalTrials.gov: NCT03149510 https://clinicaltrials.gov/ct2/show/NCT03149510		
**24) CONSORT: Where the full trial protocol can be accessed, if available**		
ClinicalTrials.gov: NCT03149510 https://clinicaltrials.gov/ct2/show/NCT03149510		
**25) CONSORT: Sources of funding and other support (such as supply of drugs), role of funders**		
"The study was funded by the National Institute on Aging at the National Institutes of Health."		
**X26-i) Comment on ethics committee approval**		
**x26-ii) Outline informed consent procedures**		
**X26-iii) Safety and security procedures**		
**X27-i) State the relation of the study team towards the system being evaluated**		
